# Anti-inflammatory components of the Vietnamese starfish *Protoreaster nodosus*

**DOI:** 10.1186/s40659-015-0002-2

**Published:** 2015-02-20

**Authors:** Nguyen Phuong Thao, Bui Thi Thuy Luyen, Jung Eun Koo, Sohyun Kim, Young Sang Koh, Nguyen Xuan Cuong, Nguyen Hoai Nam, Phan Van Kiem, Young Ho Kim, Chau Van Minh

**Affiliations:** Institute of Marine Biochemistry (IMBC), Vietnam Academy of Science and Technology (VAST), 18 Hoang Quoc Viet, Caugiay Hanoi, Vietnam; College of Pharmacy, Chungnam National University, Daejeon, 305-764 Republic of Korea; School of Medicine, Brain Korea 21 PLUS Program and Institute of Medical Science, Jeju National University, Jeju, 690-756 Republic of Korea

**Keywords:** *Protoreaster nodosus*, Starfish, IL-12 p40, IL-6, TNF-α, LPS-stimulated BMDCs, Anti-inflammatory

## Abstract

**Background:**

In the present study, we examined the inhibitory effects of a methanolic extract, dichloromethane fraction, water layer, and polyhydroxylated sterols **(1–4)** isolated from the Vietnamese starfish *Protoreaster nodosus* on pro-inflammatory cytokine (IL-12 p40, IL-6, and TNF-α) production in LPS-stimulated bone marrow-derived dendritic cells (BMDCs) using enzyme-linked immunosorbent assays (ELISA).

**Results:**

The methanolic extract and dichloromethane fraction exerted potent inhibitory effects on the production of all three pro-inflammatory cytokines, with IC_50_ values ranging from 0.60 ± 0.01 to 26.19 ± 0.64 μg/mL. Four highly pure steroid derivatives **(1–4)** were isolated from the dichloromethane fraction and water layer of *P. nodosus*. Potent inhibitory activities were also observed for (25*S*) 5α-cholestane-3β,4β,6α,7α,8β,15α,16β,26-octol **(3)** on the production of IL-12 p40 and IL-6 (IC_50s_ = 3.11 ± 0.08 and 1.35 ± 0.03 μM), and for (25*S*) 5α-cholestane-3β,6α,8β,15α,16β,26-hexol **(1)** and (25*S*) 5α-cholestane-3β,6α,7α,8β,15α,16β,26-heptol **(2)** on the production of IL-12 p40 (IC_50s_ = 0.01 ± 0.00 and 1.02 ± 0.01 μM). Moreover, nodososide **(4)** exhibited moderate inhibitory effects on IL-12 p40 and IL-6 production.

**Conclusion:**

This is the first report of the anti-inflammatory activity from the starfish *P. nodosus*. The main finding of this study is the identification oxygenated steroid derivatives from *P. nodosus* with potent anti-inflammatory activities that may be developed as therapeutic agents for inflammatory diseases.

## Background

Inflammation is one of the earliest innate immune responses to tissue injury and various pathological stimuli. It is also a critical early factor in wound healing. Delayed or impaired inflammation may affect the later stages of wound healing, especially granulation formation [1,2]. Inflammation, which represents part of the complex biological response of vascular tissue to harmful exogenous harmful stimuli, is mediated by a variety of soluble factors, including a group of secreted polypeptides known as cytokines, which play key roles in the modulation of immune responses [3].

Interleukin (IL)-12 is a pro-inflammatory cytokine produced by activated antigen-presenting cells, dendritic cells, monocytes/macrophages, and B cells in response to bacterial products and immune signals [4]. Originally identified as a B-cell differentiation factor, IL-6 is now known to be a multifunctional cytokine that participates in several biological events, including immune responses, hematopoiesis, and acute-phase reactions [5]. The regulatory effects of IL-6 involve the inhibition of tumor necrosis factor (TNF) production, providing negative feedback, and limiting acute inflammatory responses [6,7]. Cytokines such as IL-6 are essential, but their constitutive overproduction is involved in various diseases. This account for the negative regulatory mechanism in the IL-6 signaling system [8,9].

TNF has since been implicated in diverse inflammatory, infectious, and malignant conditions, and the importance of TNF in inflammation was demonstrated by the efficacy of anti-TNF antibodies or the administration of soluble TNF receptors (TNFRs) in controlling rheumatoid arthritis and other inflammatory conditions [10,11]. TNF is not typically detectable in healthy individuals, but serum and tissue levels are elevated under of inflammatory and infection [12]. Additionally, serum TNF levels correlate with the severity of infections [11]. Therefore, inhibiting the expression and production of powerful mediators, including IL-12 p40, IL-6, and TNF-α, using anti-inflammatory components could represent a preventive or therapeutic target, and may be used to develop anti-inflammatory agents for health promotion and disease prevention.

Starfish are found in all oceans. There are more than 1,500 known species, and many remain undiscovered. Forcipulatida, Paxillosida, Platyasterida, Spinulosida, and Valvatida are the main subclasses of Asteroidea. Starfish have been investigated by organic chemists, biochemists, and pharmacologists as a potential source of bioactive marine natural products. Various secondary metabolites, including steroids, steroidal glycosides, anthraquinones, alkaloids, phospholipids, peptides, and fatty acids, have been reported in starfish [13]. Steroids and their glycosylated derivatives with unique structures are known to possess anti-tumor, anti-inflammatory [14], immunomodulatory, anti-allergy, anti-fungal, hemolytic [15], neuritogenic [16], antinociceptive [17], cytotoxic [18-20], and anti-viral [21] activities.

The starfish *Protoreaster nodosus* (Linnaeus, 1758) is an invertebrate belong to the order Phanerozonia, class Asteroidea, and phylum Echinodermata. Members of the genus *Protoreaster* are found in the warm Vietnamese sea, and have been historically used as tonic agents in Vietnamese folk medicine. However, studies on the biological activities of *P. nodosus* are limited. In previous studies, the main constituents of *P. nodosus* were found to be polyhydroxylated steroids [22], several of which exhibited moderate cytotoxicity, steroidal glycoside sulfates [23], galactocerebrosides [24], and gangliosides [25].

**Figure 1:**
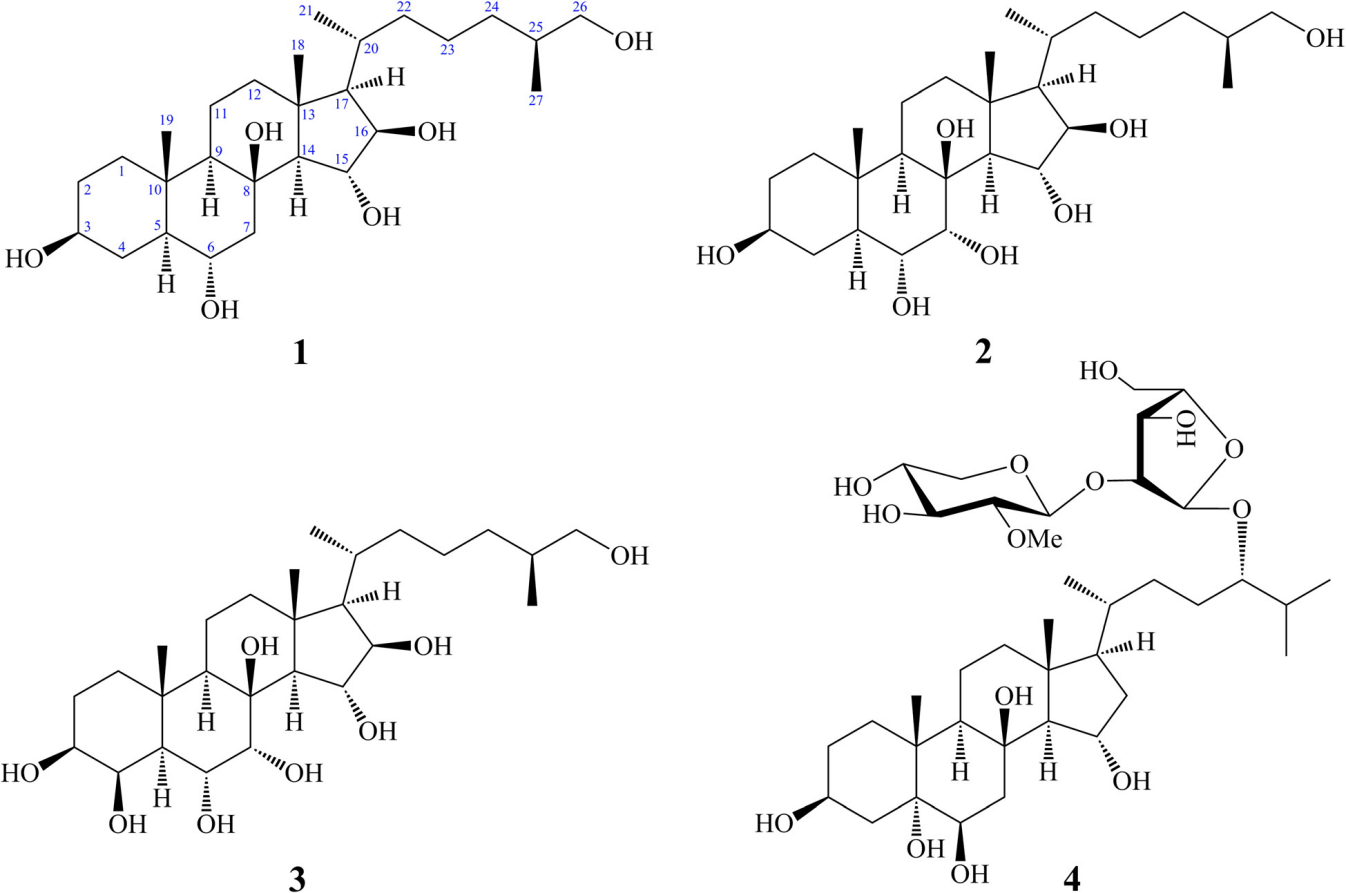
**The chemical structures of oxygenated derivatives of cholesterol (1–4) from** ***P*** ***.*** ***nodosus.***

As part of our ongoing investigations of Vietnamese marine organisms regarding anti-inflammatory activity [26-31], we found that a methanolic extract and dichloromethane fraction of *P. nodosus* showed significant *in vitro* anti-inflammatory effects. The present study details the inhibitory effect of steroid derivatives (**1**–**4**, Figure 1) from the starfish *P. nodosus* on the LPS-induced expression of the pro-inflammatory cytokines IL-12 p40, IL-6, and TNF-α in BMDCs.

## Results and discussion

Natural marine products have recently become the focus of increased research interest due to their potential pharmacological activities and low-level toxicity [32,33]. Oxysterols, or oxygenated derivatives of cholesterol produced through autooxidation or *in vivo* enzymatic processes, and have been identified in mammalian tissues and cells (e.g., blood) and processed foods. Oxysterols are emerged as intriguing substances with diverse biological activities [34,35]. Recently, it was shown that oxysterol-induced cell death shares many common features with apoptotic cell death [19], which plays an important role in the balance between cell proliferation and cell death. A wide range of stimuli can trigger cell death, which is an irreversible process [20,36].

BMDCs play a key role in the interface between the innate and acquired immune systems. Activated BMDCs perform crucial functions in immune and inflammatory responses via the pathogen-associated molecular patterns (PAMPs)-stimulated production of pro-inflammatory cytokines such as IL-12 p40, IL-6, and TNF-α, which are involved in the pathogenesis of cardiovascular and neurodegenerational diseases and cancers through a series of cytokine signaling pathways. It has been demonstrated that pro-inflammation is associated with pathophysiology and is connected with various clinical disease manifestations. These pro-inflammatory cytokines play a crucial role in host defenses and inflammation [37,38].

Among its many biological activities, IL-12 provides an obligatory signal for the differentiation of effector T-helper type 1 (Th1) cells and the secretion of Th1 cytokines, gamma interferon (IFN-γ), and IL-2. IL-12 plays an important role in the generation of Th1 responses to human pathogens [23,24]. Although the induction of IL-12 by intracellular organisms is necessary for a protective host Th1 response, the overexpression of Th1 cytokines and IL-12 may contribute to the development and perpetuation of chronic inflammatory and autoimmune diseases. Thus, understanding the regulated expression of IL-12 in macrophages may provide insight into the pathogenesis of infectious and inflammatory diseases, and could reveal novel approaches to altering immune responses [39].

To date, the anti-inflammatory effects of extracts and/or compounds isolated from the starfish *P. nodosus* have not been reported. Therefore, we assayed the anti-inflammatory activity of a methanolic extract, dichloromethane-soluble fraction, and water layer on pro-inflammatory cytokine (IL-12 p40, IL-6, and TNF-α) production in LPS-stimulated BMDCs using ELISA (Table 1). BMDCs were incubated in 48-well plates and treated for 1 h with the isolated compounds prior to stimulation with LPS (10.0 ng/mL). Supernatants were harvested 18 h after stimulation. Upon LPS treatment, dendritic cells (DCs) are known to secrete pro-inflammatory cytokines, including IL-12p40, IL-6, and TNF-α.

**Table 1:** **Anti**-**inflammatory effects of the extract**/**fractions on LPS**-**stimulated BMDCs**

**Extract**/**fractions**	**IC**_**50**_ **values** **(μg** **/mL)** ^**a**^		
	**IL** **-12 p40**	**IL-** **6**	**TNF-** **α**
CH_2_Cl_2_ fraction	0.60 ± 0.01	3.29 ± 0.09	10.29 ± 0.34
Water layer	4.03 ± 0.10	80.76 ± 1.96	>100
MeOH extract	2.48 ± 0.08	8.57 ± 0.21	26.19 ± 0.64
**SB203580** ^**b**^	2.52 ± 0.12	1.67 ± 0.13	3.65 ± 0.12

^a^The inhibitory effects are represented as giving 50% inhibition (IC_50_) relative to the vehicle control. These data represent the average values of three repeated experiments (mean ± SD). IC_50_ values for selected extracts are given in column IL-12 p40, IL-6 and TNF-α. Values <100 μg/mL are considered to be active.

^b^SB203580 was used as a positive control.

**Figure 2:**
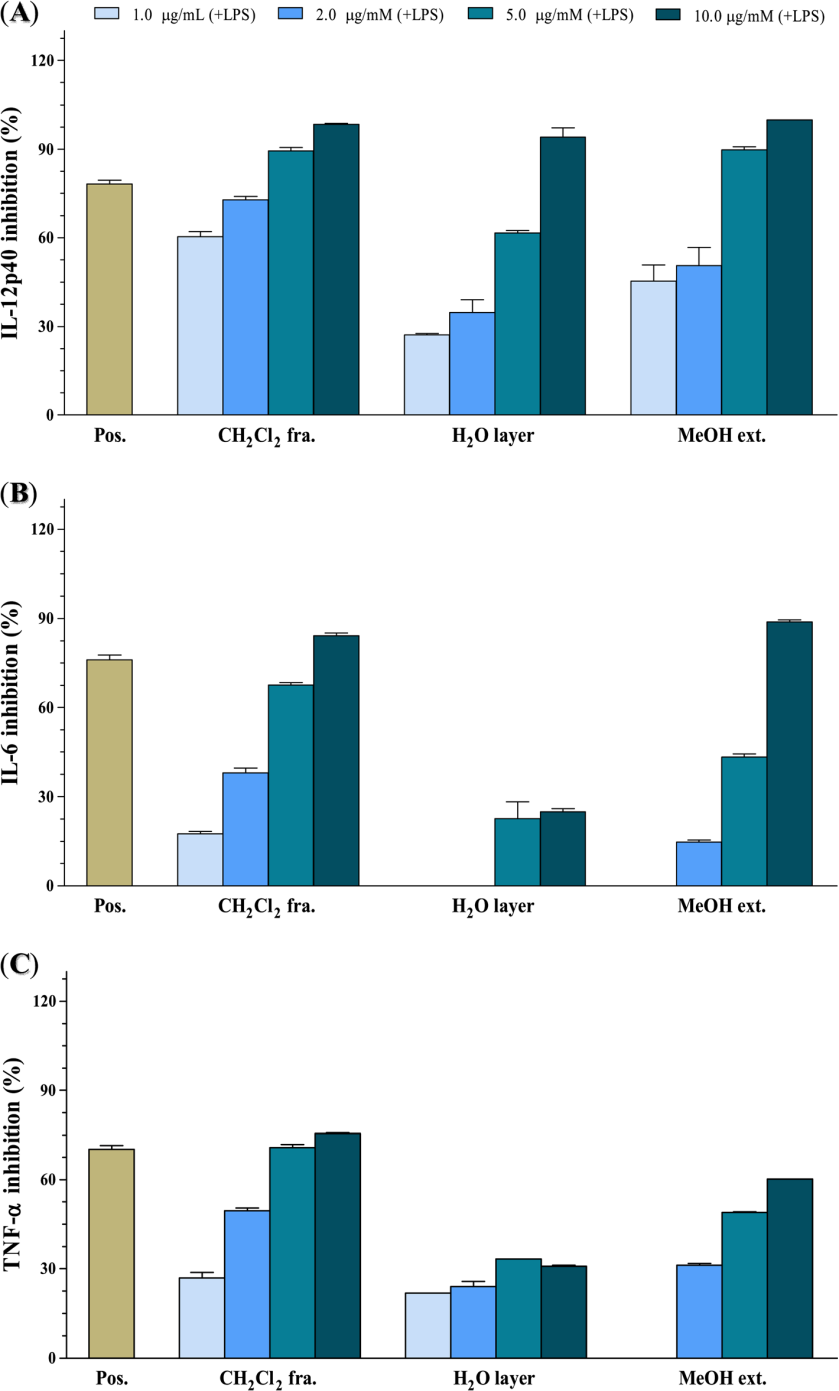
**The effect of extract** **/fractions** **(1.0,** **2.0**, **5.0,** **and 10.0 μg/** **mL**) **on IL-** **12 p40** **(A),** **IL-** **6** **(B),** **and TNF-** **α** **(C)** **production by LPS** **-stimulated BMDCs.** The data were presented as inhibition rate (%) compared to the value of vehicle-treated DCs. SB203580 was used as a positive control **(Pos.)**.

The methanolic extract significantly inhibited pro-inflammatory cytokine production, while the dichloromethane fraction and water layer showed more potent inhibitory effects (Figure 2). The methanolic extract of *P. nodosus* had inhibitory effects on IL-12 p40, IL-6, and TNF-α production (IC_50S_ = 2.48 ± 0.08, 8.57 ± 0.21, and 26.19 ± 0.64 μg/mL, respectively). Since the methanolic extract significantly reduced inflammation, it was partitioned in dichloromethane/water to obtain a dichloromethane-soluble portion and an aqueous phase. As shown in Table 1, the dichloromethane-soluble fraction showed potent inhibitory activity towards LPS-stimulated IL-12 p40 and IL-6 production (IC_50s_ = 0.60 ± 0.01 and 3.29 ± 0.09 μg/mL, respectively), which was greater than in the presence of the methanolic extract (IC_50s_ = 2.48 ± 0.08 and 8.57 ± 0.21 μg/mL). Moreover, the aqueous layer exerted potent suppressive effects on the production of IL-12 p40 (IC_50_ = 4.03 ± 0.10 μg/mL).

**Figure 3:**
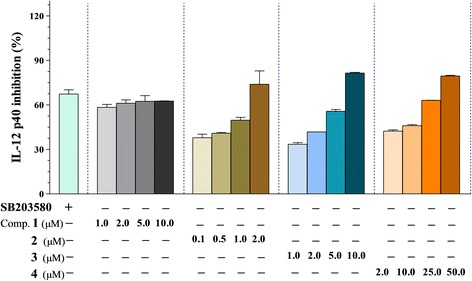
**The effect of oxygenated derivatives of cholesterol 1** **–4** **(0.1 to 50.0 μM)** **on IL-** **12 p40 production by LPS-** **stimulated BMDCs.** The data were presented as inhibition rate (%) compared to the value of vehicle-treated DCs. SB203580 was used as a positive control. Comp.: compound.

Subsequently, all of the isolated steroid derivatives **(1–4)** from the dichloromethane fraction and water layer of *P. nodosus* were tested for inhibitory effects on the production of the pro-inflammatory cytokines IL-12 p40, IL-6, and TNF-α. The results obtained with 3-(4,5-dimethyl-2,5 thiazolyl)-2,5 diphenyl tetrazolium bromide (MTT) assays showed that steroid derivatives **(1–4)** did not exhibit significant cytotoxicity at concentrations up to 50.0 μM at 24 h (data not shown). Of the tested compounds, **1–3** exerted greater inhibitory effects than SB203580 on IL-12 p40 production with IC_50_ values of 0.01 ± 0.00, 1.02 ± 0.01, and 3.11 ± 0.08 μM, respectively, at the various concentration (Figure 3 and Table 2). This variability in inflammatory response inhibition by **1–3** may be explained by the secretion of different levels of inflammatory factors upon LPS-stimulation.

**Table 2:** **Anti**-**inflammatory effects of compounds (1–4) on LPS**-**stimulated BMDCs**

**Compounds**	**IC**_**50**_ **values** **(μM)** ^**a**^		
	**IL** **-12 p40**	**IL** **-6**	**TNF-** **α**
(25*S*) 5α-Cholestane-3β,6α,8β,15α,16β,26-hexol **(1)**	0.01 ± 0.00	>100	>100
(25*S*) 5α-Cholestane-3β,6α,7α,8β,15α,16β,26-heptol **(2)**	1.02 ± 0.01	>100	>100
(25*S*) 5α-Cholestane-3β,4β,6α,7α,8β,15α,16β,26-octol **(3)**	3.11 ± 0.08	1.35 ± 0.03	>100
Nodososide **(4)**	12.47 ± 0.38	23.18 ± 0.46	>100
**SB203580** ^**b**^	5.00 ± 0.16	3.50 ± 0.12	7.20 ± 0.13

^a^The inhibitory effects are represented as giving 50% inhibition (IC_50_) relative to the vehicle control. These data represent the average values of three repeated experiments (mean ± SD). IC_50_ values for selected extracts are given in column IL-12 p40, IL-6 and TNF-α. Values <100 μM are considered to be active.

^b^SB203580 was used as a positive control.

Comparing the observed effects between the structurally steroid derivatives, (25*S*) 5α-cholestane-3β,6α,8β,15α,16β,26-hexol **(1)** is similar to that of (25*S*) 5α-cholestane-3β,6α,7α,8β,15α,16β,26-heptol **(2)** and (25*S*) 5α-cholestane-3β,4β,6α,7α,8β,15α,16β,26-octol **(3)**, except for the presence of hydroxy groups located at C-4 and/or C-7 in compounds **2** and **3**; these compounds had the greatest inhibitory activity towards LPS-stimulated IL-12 p40 production (IC_50_ = 0.01 ± 0.00 μM), which was comparable to that of the positive control, SB203580 (IC_50_ = 5.00 ± 0.16 μM). Previously, compounds **1–3** were isolated from *Asterina pectinifera, Pentaceraster alveolatus*, and *Oreaster reticulatus* [40-42], and they were evaluated for their antiviral activity against herpes simplex virus type 1 (HSV-1) and their cytotoxicity against human liver carcinoma (HepG2) cells *in vitro* [40]. Nodososide **(4)** is a polyhydroxylated steroid glycoside. Structurally, these glycosides are oxygenated derivatives of cholesterol that are glycosylated with a disaccharide chain, but **4** exhibited a moderate effect (IC_50_ = 12.47 ± 0.38 μM) up to 50.0 μM, relative to the vehicle group. Our finding that steroid derivatives **(1–3)** possess significant anti-inflammatory activity suggests that these related steroids might be interesting for further investigations for potential inflammatory diseases.

**Figure 4:**
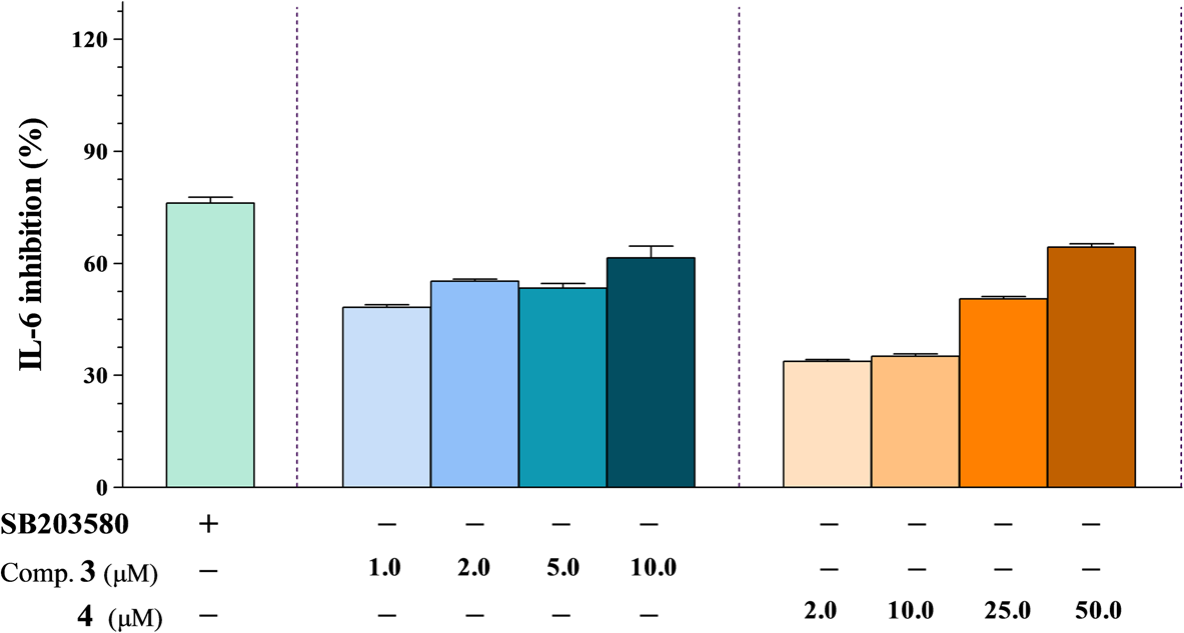
**The effect of oxygenated derivatives of cholesterol 3 and 4** **(1.0 to 50.0 μM)** **on IL-** **6 production by LPS-** **stimulated BMDCs.** The data were presented as inhibition rate (%) compared to the value of vehicle-treated DCs. SB203580 was used as a positive control. Comp.: compound.

IL-6 is a pro-inflammatory cytokine that induces inflammatory upon secretion by T cells and macrophages in response to trauma, especially burns or other types of tissue damage [43]. It also strongly activates the immune system and enhances inflammatory responses, although based on some of its effects, it may also be classified as anti-inflammatory cytokine. Regarding IL-6, the methanolic extract and dichloromethane-soluble fraction showed inhibitory ciliary neurotropic factor activity toward LPS-stimulated IL-6 production (IC_50s_ = 8.57 ± 0.21 and 3.29 ± 0.09 μg/mL, respectively). (25*S*) 5α-cholestane-3β,4β,6α,7α,8β,15α,16β,26-octol **(3)** inhibited IL-6 production significantly in LPS-stimulated BMDCs, with an IC_50_ value of 1.35 ± 0.03 μM (Table 2). The inhibitory effects of nodososide **(4)** included moderate inhibition of IL-6 production, with an IC_50_ value of 23.18 ± 0.46 μM (Figure 4). The remaining compounds did not show significant activity (IC_50_ > 100 μM) against IL-6 production.

Overexpression of the pro-inflammatory cytokines TNF-α and IL-6 is associated with the development of autoimmune, inflammatory, and immunopathological diseases. Therefore, blocking TNF-α, IL-6, and their respective signaling pathways may be effective for the treatment of inflammatory diseases. According to our results, the methanolic extract and dichloromethane-soluble fraction showed inhibitory effects on LPS-stimulated TNF-α production (IC_50s_ = 26.19 ± 0.64 and 10.29 ± 0.34 μg/mL, respectively). Notably, not all of the isolated steroid derivatives **(1–4)** had significant inhibitory effects on TNF-α production (IC_50_ > 100 μM, Table 1 and 2). However, it is interesting to note that the methanolic extract extract and dichloromethane-soluble fraction of *P. nodosus* were more active than the isolated steroid derivatives **(1–4)**, suggesting the presence of other active derivatives, which could act individually or by synergy with the isolated steroid derivatives **(1–4)**.

## Conclusion

In conclusion, the methanolic extract and dichloromethane fraction showed potent inhibitory effects on the production of all three pro-inflammatory cytokines, with IC_50_ values ranging from 0.60 ± 0.01 to 26.19 ± 0.64 μg/mL. Potent inhibitory activities were observed for (25*S*) 5α-cholestane-3β,4β,6α,7α,8β,15α,16β,26-octol **(3)** on the production of IL-12 p40 and IL-6, and for (25*S*) 5α-cholestane-3β,6α,8β,15α,16β,26-hexol **(1)** and (25*S*) 5α-cholestane-3β,6α,7α,8β,15α,16β,26-heptol **(2)** on the production of IL-12 p40. Moreover, nodososide **(4)** exerted moderate inhibitory effects on IL-12 p40 and IL-6 production. Additional studies are required to assess the mechanisms of action of these compounds and their potential use as novel anti-inflammatory agents. These results support the use of starfish steroid components to inhibit the secretion of pro-inflammatory cytokines, including IL-12 p40, IL-6, and TNF-α, and to prevent and treat inflammatory diseases.

## Methods

### General experimental procedures

Optical rotations were determined on a JASCO P-2000 polarimeter. The UV spectrum was recorded on a JASCO V-630 spectrophotometer. IR spectra were obtained on a Bruker TENSOR 37 FT-IR spectrometer. The ^1^H NMR (500 MHz) and ^13^C NMR (125 MHz) spectra were recorded on a Bruker AM500 FT-NMR spectrometer and TMS was used as an internal standard. Column chromatography (CC) was performed on silica gel (Kieselgel 60, 70–230 mesh and 230–400 mesh, Merck), porous polymer gel (Mitsubishi Chemical, Diaion HP-20, 70 × 180 mm), octadecyl silica (ODS, Cosmosil 140 C_18_-OPN, Nacalai Tesque), and YMC RP-18 resins (30–50 μm, Fuji Silysia Chemical). Thin layer chromatography (TLC) used pre-coated silica gel 60 F_254_ (1.05554.0001, Merck) and RP-18 F_254S_ plates (1.15685.0001, Merck) and compounds were visualized by spraying with aqueous 10% H_2_SO_4_ and heating for 3–5 minutes.

### Marine material

The sample of the starfish *P. nodosus* was collected at Ha Long, Quangninh, Vietnam, in October 2012 and identified by Prof. Do Cong Thung (Institute of Marine Resources and Environment, VAST, Hanoi, Vietnam). A voucher specimen (PN-VHS-2012) was deposited at the Institute of Marine Resources and Environment and Institute of Marine Biochemistry, VAST, Vietnam.

### Extraction and isolation

Fresh frozen samples of the starfish *P. nodosus* (5.0 kg) were ground and extracted three times with hot MeOH (at 50°C for 3 h each time). The obtained solutions were filtered, combined, and concentrated under reduced pressure to yield a dark viscous residue (56.0 g, A). This residue was suspended in water (1.0 L) and partitioned in turn with CH_2_Cl_2_ (3 × 1.0 L). The combined dichloromethane-soluble portions were evaporated under reduced pressure to afford CH_2_Cl_2_ fraction (27.5 g, B) and water layer (C).

Extract B was crudely separated by silica gel (CC) using gradient concentrations of ethyl acetate (EtOAc) in *n*-hexane from 0 to 100% to yield four fractions (B-1 to B-4). Fraction B-4 (2.31 g) was further separated by silica gel CC using CH_2_Cl_2_-MeOH-H_2_O (5:1:0.1) as eluents, to give three subfractions (B-1.1 to B-1.4). Subfraction B-1.3 (1.35 g) was then subjected to silica gel CC using eluent of CH_2_Cl_2_-MeOH (3.5:1), and further purified by reversed-phase flash CC (YMC Gel ODS-A, 60 Å, 400/500 mesh) and eluting with MeOH-H_2_O (2:1) to afford (25*S*) 5α-cholestane-3β,6α,8β,15α,16β,26-hexol (**1**, 2.1 mg). Next, (25*S*) 5α-Cholestane-3β,6α,7α,8β,15α,16β,26-heptol (**2**, 1.8 mg) was purified via the subfraction of B-1.2 (0.21 g) by silica gel CC and eluting with CH_2_Cl_2_-MeOH (4.5:1). Similarly, subfraction B-1.1 (4.16 g) was subjected to YMC RP-18 CC, using MeOH-acetone-H_2_O (80:10:10) to yield (25*S*) 5α-cholestane-3β,4β,6α,7α,8β,15α,16β,26-octol (**3**, 2.3 mg).

The water layer was desalted by Diaion™ HP-20 CC and eluted first with water and then with MeOH. The desalted water residue (6.2 g, C) was separated by silica gel CC and eluted with CH_2_Cl_2_-MeOH (from 25:10:1, v/v/v) to yield five fractions (C-1 to C-5). Fraction C-3 (1.2 g) was separated by silica gel CC, using CH_2_Cl_2_-MeOH (5:1) as the eluent to yield three subfractions (C-3.1 to C-3.3). Finally, subfraction C-3.3 (0.15 g) was further separated by Sephadex® LH-20 CC using acetone-MeOH (1:2) as the mobile phase, followed by YMC RP-18 CC using MeOH-H_2_O (1:1) to afford nodososide (**4**, 3.5 mg).

### Marine product derivatives

In total, 5.0 kg of finely chopped material were extracted with MeOH (5.0 L) in glass jars with screw-on lids. The methanolic extracts were filtered, combined, and concentrated using a rotary vacuum evaporator at 40°C and then further dried *in vacuo* at ambient temperature for 24 h. From the dichloromethane fraction and water layer of *P. nodosus*, four highly pure steroid derivatives **(1–4)** were isolated and their structures elucidated. Stock solutions of the tested compounds were prepared in dimethyl sulfoxide (DMSO), kept at −20°C, and diluted to the desired final concentration in fresh medium before each experiment. The final concentration of DMSO did not exceed 0.5% in any of the experiments to avoid effects cell growth.

### Cell viability assay

The cell viability was determined by standard procedure of MTT assays (Sigma, St. Louis, MO, USA), which was based on the reduction of the dye MTT to formazan crystals, an insoluble intracellular blue product, by cellular dehydrogenases. Briefly, the cells at a concentration of 5 × 10^4^ cells were seeded on a 96-well culture plate. After incubation for 1 h at 37°C, cells were treated with extract, fractions, and compounds at various concentrations for 24 h. Cells were added 0.2 mg MTT (Sigma, St. Louis, MO) and then incubated at 37°C for 4 h. The plate was centrifuged and the supernatants were aspirated. The formazan crystals in each well were dissolved in 250 μL DMSO (Amresco, OH). The plates were agitated to ensure complete dissolution of the purple formazan crystals, and the optical density was measured at the wavelength 540 nm using an ELISA reader (Packard, Instrument Co., Downers Grove, IL).

### Cell cultures

In this study, we used LPS-stimulated BMDCs as a model for testing the inhibitory effects of fractions and isolated compounds on the secretion of pro-inflammatory cytokines IL-12 p40, IL-6, and TNF-α. BMDCs (1 × 10^5^) were seeded in 48-well plates at 37°C, 5% CO_2_ for 1 h, and then treated for 1 h with the compounds at concentrations of 0.1 to 50.0 μM, followed by stimulation with LPS (10.0 ng/mL, Figure 3). The supernatants were harvested 18 h after stimulation and IL-12 p40 secretion was measured using ELISA. SB203580, an inhibitor of cytokine suppressive binding protein/p38 kinase, was used as a positive control [44]. SB203580 inhibited IL-12 p40, IL-6, and TNF-α production with IC_50_ values of 5.00 ± 0.16, 3.50 ± 0.12, and 7.20 ± 0.13 μM, respectively.

Bone marrow-derived dendritic cells were grown from wild-type C57BL/6 mice (Orient Bio Inc., Seoul, Korea) as previously described [45]. All animal procedures were approved by and performed according to the guidelines of the Institutional Animal Care and Use Committee of Jeju National University (#2010-0028). Briefly, the mouse tibia and femur was obtained by flushing with Dulbecco’s modified Eagle medium to yield bone marrow cells. The cells were cultured in Rosell Park Memorial Institute (RPMI) 1640 medium containing 10% heat-inactivated fetal bovine serum (FBS; Gibco, Grand Island, NY, USA), 50.0 μM β-mercaptoethanol, and 2.0 mM glutamine supplemented with 3% J558L hybridoma cell culture supernatant containing granulocyte-macrophage colony-stimulating factor (GM-CSF). The culture medium was replaced with fresh medium every other day. At day six of culture, non-adherent cells and loosely adherent dendritic cell (DC) aggregates were harvested, washed, and resuspended in RPMI 1640 supplemented with 5% FBS.

### Cytokine production measurements

The BMDCs were incubated in 48-well plates in 0.5 mL containing 1 × 10^5^ cells per well, and then treated with the isolated compounds **(1–4)** at different concentrations for 1 h before stimulation with 10.0 ng/mL LPS from *Salmonella minnesota* (Alexis, Famingdale, NY, USA). Supernatants were harvested 18 h after stimulation. Concentrations of murine IL-12 p40, IL-6, and TNF-α, in the culture supernatant were determined by ELISA (BD PharMingen, San Diego, CA, USA) according to the manufacturer’s instructions. The data are presented as means ± S.D. of at least three independent experiments performed in triplicate.

IL-12 p40 level in unstimulated DC: not detectable. IL-12 p40 level in LPS-stimulated DC: 51.34 ± 0.66 (ng/mL). IL-6 level in unstimulated DC: not detectable. IL-6 level in LPS-stimulated DC: 41.12 ± 2.38 (ng/mL). TNF-α level in unstimulated DC: not detectable. TNF-α level in LPS-stimulated DC: 1.83 ± 0.02 (ng/mL).

The inhibitory activity (I) was expressed as the inhibition rate (%), which was calculated from the following formula:

$$ I=\frac{C_{\mathrm{dcv}}-{C}_{\mathrm{dcc}}}{C_{\mathrm{dcv}}}\times 100 $$

*C*_dcv_: Cytokine level (ng/mL) in vehicle treated DC; *C*_dcc_: Cytokine level (ng/mL) in compound treated DC.

### Statistical analysis

The results are expressed as mean value ± S.D. Statistical analysis was performed using one-way ANOVA. *P* < 0.05 was considered statistically significant.
